# Methylene blue prevents osteoarthritis progression and relieves pain in rats via upregulation of Nrf2/PRDX1

**DOI:** 10.1038/s41401-021-00646-z

**Published:** 2021-04-08

**Authors:** Jia-wei Li, Rong-liang Wang, Jia Xu, Kuo-yang Sun, Hui-ming Jiang, Zi-ying Sun, Zhong-yang Lv, Xing-quan Xu, Rui Wu, Hu Guo, Qing Jiang, Dong-quan Shi

**Affiliations:** 1 State Key Laboratory of Pharmaceutical Biotechnology, Department of Sports Medicine and Adult Reconstructive Surgery, Affiliated Drum Tower Hospital, Medical School of Nanjing University, Nanjing, 210008 China; 2 Drum Tower of Clinical Medicine, Nanjing Medical University, Nanjing, 210008 China; 3 Department of Sports Medicine and Joint Surgery, the Affiliated Nanjing Hospital of Nanjing Medical University, Nanjing, 210008 China

**Keywords:** methylene blue, osteoarthritis, oxidative stress, carilage protection, pain relief

## Abstract

Oxidative stress-related cartilage degeneration, synovitis, and joint pain play vital roles in the progress of osteoarthritis (OA). Anti-oxidative stress agents not only prevent structural damage progression but also relieve OA-related pain. In this study, we investigated the therapeutic effect of methylene blue (MB), a classical and important anti-oxidant with strong neural affinity. Experimental OA was established in rats by radial transection of medial collateral ligament and medial meniscus (MCLT + MMT) of the right knee joint. The OA rats received intra-articular injection of MB (1 mg/kg) every week starting one week after surgery. We showed that MB administration exerted significant cartilage protection, synovitis inhibition as well as pain relief in OA rats. In human chondrocytes and fibroblast-like synoviocytes, MB significantly attenuated tert-butyl hydroperoxide (TBHP)-induced inflammatory response and oxidative stress. We demonstrated that these effects of MB resulted from dual targets of important antioxidant enzymes, Nrf2 and PRDX1, which also mutually reinforcing and participated in an interaction. Furthermore, we found that calcitonin gene-related peptide (CGRP), a neural inflammatory mediator, was accumulated around the vessel in synovium and subchondral bone in OA rats and in TBHP-treated primary cortical neurons; MB administration significantly inhibited CGRP expression through upregulation of Nrf2 and PRDX1. Taken together, these results suggest that MB ameliorates oxidative stress via Nrf2/PRDX1 regulation to prevent progression and relieve pain of OA.

## Introduction

Osteoarthritis (OA) is a painful and disabling condition involving chronic joint degeneration that affects millions of people and imposes an enormous burden on the health care system worldwide [1, 2]. Pain is a major symptom of OA that becomes more severe and frequent with OA progression [3]. Currently, despite the side effects related to their prolonged use, NSAIDs are the primary effective agents for relieving pain [4]. Strategies for effectively relieving pain are lacking. As cartilage is not innervated, cartilage damage rarely leads directly to pain [3]. However, several structural changes associated with cartilage loss, including synovial thickening (synovitis), bone marrow lesions, and knee effusion, have been demonstrated to be related to the development of pain [5–7]. Attempts to identify drugs that can prevent structural damage progression and relieve pain associated with joint damage are ongoing [8].

Inflammation resulting from aging and/or mechanical overload leads to an increase in oxidative stress, the accumulation of reactive oxygen species (ROS), hydrogen peroxide (H_2_O_2_), and superoxide anions, and interference with the expression of antioxidant enzymes and molecules involved in ROS scavenging systems [9, 10]. Methylene blue (MB) was initially discovered as a dye with a strong affinity for nerve endings [11] and has subsequently been used as a long-term inhibitor of peripheral nerve axons [12–14]. MB is an important antioxidant and anti-inflammatory agent that can be used to treat clinical pain syndromes, cyanide poisoning, and neurodermatitis [12, 15, 16]. MB may have the potential to relieve pain and protect the joint structure in OA.

The regulation of ROS scavenging systems and antioxidant enzymes plays a key role in suppressing pain and the progression of OA. Nuclear factor (erythroid-derived 2)-like 2 (Nrf2) is an important cellular redox regulator that participates in the regulation of protective factors involved in the recognition and clearance of damaged proteins and organelles [17, 18]. The regulation of oxidative stress and inflammation by Nrf2 protects against joint alterations in OA. The protective effects of MB in upregulating Nrf2 expression to prevent tau-related neurotoxicity have been studied in the context of neurodegenerative diseases [19], but the effects of MB in OA and especially in relieving pain in OA have not yet been studied. Peroxiredoxins (PRDXs), which are members of the Nrf2-dependent phase II gene family, are classified according to the presence of one (1-Cys) or two (2-Cys) conserved cysteine residues [20–22]. As a major member of this antioxidant family, PRDX1 is mainly located in the cytosol, is involved in interactions with several ROS-dependent effectors and plays a key intracellular role in maintaining cellular survival and metastasis [21]. Most studies on PRDX1 have focused on its antioxidant effect in cancer [21], and only a few studies have investigated its role in acute neural injury, such as spinal cord injury [23]. As two major antioxidant stress enzymes, Nrf2 and PRDX1 interact with each other and form a network in healthy and diseased tissues and cells [24].

Herein, we utilized tert-butyl hydroperoxide (TBHP) as an exogenous agent to induce oxidative stress in chondrocytes, synoviocytes, and neurons. TBHP has been widely used for in vitro studies of ROS in OA [25–27] and possesses advantages over H_2_O_2_ such as slow-release and high stability [28]. Then, we investigated the effect of MB in regulating ROS in a rat model of OA and demonstrated the potential mechanism of the Nrf2/PRDX1 interaction. These findings demonstrate the promising therapeutic effect of MB on OA and suggest that Nrf2/PRDX1 is an effective therapeutic target for relieving pain and alleviating OA.

## Materials and methods

### Ethics statement

The protocols used for the collection and analysis of human articular cartilage and synovium were approved by the Ethical Committee of the Nanjing Drum Tower Hospital, the Affiliated Hospital of Nanjing University Medical School (2009022). All experimental procedures followed the guidelines of the Declaration of Helsinki [29]. All animal surgery, treatment, and postoperative care procedures were performed in strict accordance with the guidelines of the Animal Care and Use Committee of Nanjing Drum Tower Hospital, the Affiliated Hospital of Nanjing University (2018020005).

### Animal study

Adult male Sprague-Dawley (SD) rats (220–250 g, *n* = 30) were acquired from the Animal Center of Nanjing Medical University (Jiangsu, China) and acclimated for 1 week before the operation. After acclimation, twenty rats underwent radial transection of the medial collateral ligament (MCLT) and medial meniscus (MMT) (MCLT + MMT), and ten rats underwent sham operation. After surgery, the rats were randomly divided into three groups: the sham group, OA group, and OA with MB treatment group. Following transection of the MCLT + MMT, 1 mg/kg MB (Sigma-Aldrich, Darmstadt, Germany) was intra-articularly injected into the rats in the OA with MB treatment group each week, and physiological saline was injected into the rats in the OA group. The treatment started one week after surgery and continued until the rat sacrificed.

### Pain sensitivity test

We investigated differences in the nociceptive responses of rats to mechanical stimuli with an electronic von Frey Anesthesiometer (IITC, Woodland Hills, USA). Before each test, the animals were placed in transparent Plexiglas compartments (20 cm long, 25 cm wide, and 15 cm high) for half an hour for habituation. The compartments were placed on a metal mesh floor, which allowed the tip of the anesthesiometer to stimulate the mid-plantar region of the affected hind paw. All tests were repeated at least five times with an interval of at least ten minutes between stimulations. The observer was blinded to the force applied in the test. The paw withdrawal mechanical threshold (PWMT) of each rat was measured as the tolerance level in grams, and the mean PWMT was calculated by averaging the results of the final three tests. The test was performed every week after MCLT + MMT transection.

### Measurement of hindlimb weight distribution

Hindlimb weight distribution was measured with a hindlimb weight meter (Kew Instrumentation, kw-11A) as an indicator of pain. The rats were positioned on a capacitance meter with their hindlimbs resting on two separate force sensors. After at least 10 min of habituation, the weight on each force plate was recorded. The observer was blinded to the weights measured in the test. Then, the ratio of the value for the affected limb to the total value for both hindlimbs was calculated as the weight distribution of the affected limb to reflect the degree of OA-related pain. The test was performed each week after MCLT + MMT.

### OA severity assessment

Assessment of OA severity, including measurement of the affected knee joint diameter and gait analysis, was performed before surgery and 6 weeks after surgery. The knee joint diameter was defined as the maximum length of the coronal plane of the rat knee joint and was measured with a Vernier caliper (the distance from the medial femoral condyle to the lateral condyle plus the thickness of the swollen joint synovium). Gait analysis was performed to assess the claudication of the affected limb induced by OA. Briefly, the animals were placed in a 100 × 10 cm open gait arena and allowed to freely walk from one side to the other in the absence of an external stimulus or food enticement. The footprints of each rat were recorded by dipping the animal’s hind paws with red dye and forepaws with blue dye. Outcome measures were obtained by three independent examiners who were blinded to the experimental conditions.

### Micro-computed tomography (micro-CT)

Rat knee joints were fixed in 4% PFA, and then the microstructure of the joints was analyzed using a micro-CT scanner (mCT80; Scanco Medical AG). The scanner was set at a voltage of 70 kV, a current of 114 μA, and a resolution of 15.6 μm per pixel. 3D reconstruction images were acquired with Scanco Medical software.

### Cell culture

Five human OA cartilage samples were collected from OA patients during total knee arthroplasty (54–74 years old; Kellgren–Lawrence grade IV; *n* = 5). For primary human chondrocyte (hCH) culture, fresh cartilage was cut into 1 mm^3^ cubes and washed with phosphate-buffered saline (PBS) under sterile conditions. Then, the cartilage cubes were lysed with 0.2% collagenase II in Dulbecco’s modified Eagle’s medium/nutrient mixture F12 (DMEM/F12) at 37 °C for 6 h. For primary human fibroblast-like synoviocyte (hFLS) culture, the synovium was cut into 1 mm^3^ fragments and washed with PBS under sterile conditions. Subsequently, the synovial fragments were lysed with 0.2% collagenase I in DMEM/F12 at 37 °C for 6 h. After filtration and centrifugation, the pellet was placed in DMEM/F12 supplemented with 10% fetal bovine serum. The cells were cultured in a humid environment at 37 °C and 5% CO_2_ and the medium was replaced every 2 days.

### Cell viability assay

To assess the cytotoxicity of TBHP to chondrocytes and synoviocytes treated with or without MB, the cell counting kit-8 (CCK-8) assay (Dojindo Co, Kumamoto, Japan) was performed according to the manufacturer’s protocol. An equal number of hCHs and hFLSs were seeded in 96-well plates and then treated with TBHP and/or MB for 6 h. After being washed with PBS, the cells in each well were incubated with DMEM/F12 comprising 10%(v/v) CCK-8 solution at 37 °C for 2 h. The absorbance was measured at 450 nm with a microplate reader (Thermo Scientific, Logan, UT, USA).

### Western blot analysis

Protein was extracted from hCHs and hFLSs with RIPA lysis buffer supplemented with 1 mM phenylmethanesulfonyl fluoride and 1 mM protein phosphatase inhibitor and then centrifuged for 10 min at 12,000 r/min at 4 °C. A BCA protein assay kit (Thermo Scientific) was used to measure the protein concentration. The proteins were separated on 10% (w/v) SDS-polyacrylamide gels and transferred onto polyvinylidene fluoride membranes (Bio-Rad, Hercules, CA, USA). The membranes were blocked with 5% (w/v) milk (Bio-Rad) for 2 h at room temperature and then incubated overnight at 4 °C with primary antibodies against MMP1 (1:1000, Proteintech, Wuhan, China), MMP3 (1:1000, Proteintech), MMP13 (1:1000, Cell Signaling Technology, Danvers, USA), Nrf2 (1:1000, Cell Signaling Technology), Keap1 (1:2000, Proteintech), β-actin (1:1000, Proteintech), Histone H3 (1:1000, Cell Signaling Technology), HO-1 (1:500, Proteintech), NQO1 (1:500, Proteintech), PRDX1 (1:1000, Proteintech), Collagen II (1:2000, Abcam, Cambridge, UK), SOX9 (1:1000, Cell Signaling Technology), and GAPDH (1:2000, Cell Signaling Technology). Next, the membranes were washed with TBS with 0.05% Tween 20 (TBST) three times and incubated with horseradish peroxidase-conjugated secondary antibodies for 60 min. The signals were detected with a ChemiDocXRS + Imaging System (Tanon, Shanghai, China). All experiments were repeated 5 times.

### Quantitative real-time PCR

Cellular mRNA was isolated and cDNA was generated by quantitative real-time PCR (qRT-PCR) essentially as described previously [30]. The primer sequences were as follows:

*IL-1β*-F: 5′-ATGATGGCTTATTACAGTGGCAA-3′; *IL-1β*-R: 5′-GTCGGAGATTCGTAGCTGGA-3′

*IL-6*-F: 5′-CCTGAACCTTCCAAAGATGGC-3′; *IL-6*-R: 5′-TTCACCAGGCAAGTCTCCTCA-3′

*NOX4*-F: 5′-TGACGTTGCATGTTTCAGGAG-3′; *NOX4*-R: 5′-AGCTGGTTCGGTTAAGACTGAT-3′

*SOD1*-F: 5′-GGTGGGCCAAAGGATGAAGAG-3′; *SOD1*-R: 5′-CCACAAGCCAAACGACTTCC-3′

*SOD2*-F: 5′-GCTCCGGTTTTGGGGTATCTG-3′; *SOD2*-R: 5′-GCGTTGATGTGAGGTTCCAG-3′

and *GAPDH*-F: 5′-ACAACTTTGGTATCGTGGAAGG-3′; *GAPDH*-R: 5′-GCCATCACGCCACAGTTTC-3′.

### Measurement of intracellular ROS levels

hCHs and hFLSs were plated in six-well plates (2.0 × 10^5^ cells/well) and then treated with TBHP (Sigma-Aldrich, Darmstadt, Germany) with or without MB (Sigma-Aldrich, Darmstadt, Germany). Next, the cells were stained with 10 mmol/L 2′,7′-dichlorodihydrofluorescein diacetate (DCFH-DA) (Beyotime, Shanghai, China) for 30 min at 37 °C. ROS levels were determined under a fluorescence microscope.

### Histopathologic analysis

Six weeks after surgery, the SD rats were sacrificed, and the affected knee joints were collected. The knee joints were fixed in 4% (v/v) paraformaldehyde for 1 day and then decalcified in 10% (v/v) EDTA for 2 months. After dehydration, the specimens were embedded in paraffin and cut into 3 μm coronal sections. The tissue sections were stained with safranin O-fast green (SO) and hematoxylin and eosin (H&E). The Osteoarthritis Research Society International scoring system was used to evaluate the medial femoral condyles and medial tibial plateaus of the rats in each group, and the severity of synovitis and the formation of osteophytes were graded using a scoring system as previously described [31].

### Immunohistochemical staining

After deparaffinization and rehydration, endogenous peroxidase activity in the sections was blocked with 3% hydrogen peroxide. Then, for antigen retrieval, the sections were incubated with 0.4% pepsin (Sigma-Aldrich) in 1 mM hydrochloric acid at 37 °C for 1 h. After blocking with 5% bovine serum albumin for 30 min at 37 °C, the sections were incubated with primary antibody overnight at 4 °C and finally with an HRP-conjugated secondary antibody.

### Cell immunofluorescence

Cells were washed with PBS, fixed in 4% paraformaldehyde, and permeabilized with 0.1% Triton X-100 for 15 min. After blocking with 5% bovine serum albumin for 1 h at 37 °C, the cells were incubated with primary antibodies overnight at 4 °C. The cells were washed with PBS and incubated with FITC- or TRITC-conjugated secondary antibodies for 1 h at 37 °C and stained with DAPI for 7 min. Twenty fields from each slide were randomly chosen for observation under a fluorescence microscope (Zeiss Inc., Heidelberg, Germany).

### Short interfering RNA (siRNA) transfection

A short interfering RNA (siRNA) against the human PRDX1 gene was designed and synthesized (Hippobio, Zhejiang, China); the sequence of the siRNA was 5'-UUCUGCCCUAUCACUGAAAGCTT-3'. We plated chondrocytes and synoviocytes in six-well plates and cultured them to ~70% confluence. Then, the cells were transfected with 50 nM siRNA-PRDX1 or negative control with Lipofectamine 3000 for 12 h (Thermo Fisher, Logan, UT, USA) according to the manufacturer’s instructions.

### Primary neuron culture

Primary cortical neurons isolated from P1 SD rats were first plated on poly-D-lysine-coated glass coverslips in DMEM/F12 supplemented with 10% [v/v] FBS. Four hours after plating, the adherent cell culture medium was replaced with neurobasal medium supplemented with 2% (v/v) B27 and 1% *L*-glutamine. When the neurons reached ~60%–70% confluence, they were treated with TBHP and with or without MB.

### Statistical analysis

All data are expressed as the means ±S.D.s. All data were statistically analyzed by one-way analysis of variance (ANOVA) using GraphPad Prism 8. Differences were considered statistically significant when *P* < 0.05.

## Result

### MB relieves pain and ameliorates joint deterioration in OA

**Fig. 1:**
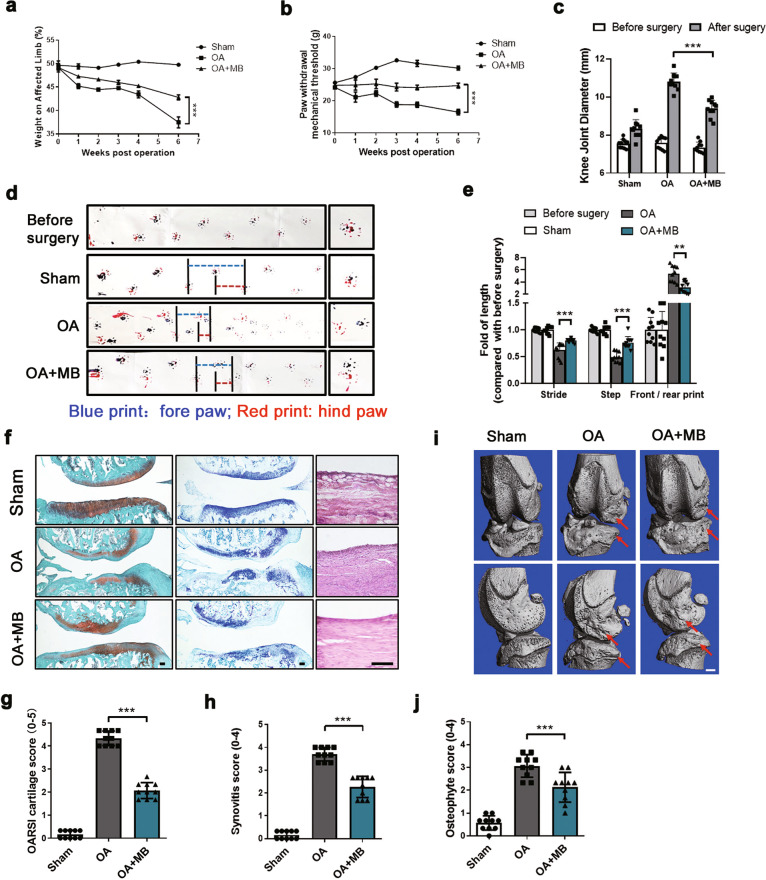
MB relieves pain and ameliorates joint deterioration in OA. **a** Changes in weight distribution on the affected limbs of the rats every week after surgery; *n* = 10. **b** Changes in pain sensitivity every week after surgery, as determined by measuring the PWMT; *n* = 10. **c** Knee joint diameter of the affected limbs of the rats before surgery and 6 weeks after surgery. **d** Gait analysis results for the different groups. The blue dotted line represents stride length; the red dotted line represents step length. Blue print: forepaw; red print: hind paw. **e** Quantification of stride length, step length, and the length of the front/rear paw prints; *n* = 10. **f** Safranin O and toluidine blue staining of cartilage and H&E staining of the synovium in different groups 6 weeks after surgery (scale bar: 100 μm). **g** The OARSI scores of cartilage samples from the different groups. **h** The synovitis scores of samples from the different groups. **i** Micro-CT analysis of knee joints from the different groups. **j** The osteophyte scores of samples from the different groups. All data represent mean ± S.D. *P < 0.05, **P < 0.01, and ***P < 0.001.

In this study, surgery was performed to remove the medial meniscus from the right knee joints of SD rats to establish an OA model. One week after the operation, MB (1 mg/kg) was injected into the right articular joint of each rat in the OA + MB group every week. As shown in Fig. 1a, weight distribution on the right limb was maintained at ~50% before surgery, which represented a normal baseline, in the sham operation group and the surgery groups. Weight distribution on the operated limb was <50% in the OA group and the OA + MB group after surgery, illustrating that the rats exhibited pain in the right leg after modeling. Weight distribution on the operated leg in the OA + MB group was higher than that in the OA group, indicating that MB relieved pain during the development of OA. Furthermore, we investigated changes in nociceptive responses to mechanical stimuli after surgery by performing a pain sensitivity test and quantifying the paw withdrawal mechanical threshold (PWMT) every week after surgery (Fig. 1b). The OA group and OA + MB group presented significantly lower PWMTs than the sham operation group, indicating that OA induced hyperalgesia in the animals. However, the rats in the OA + MB group exhibited higher PWMTs than those in the OA group. These results indicated that intra-articular MB injection markedly alleviated persistent mechanical allodynia. In addition, we measured the maximal coronal diameter (medial and lateral) of the affected knee joint with Vernier calipers to assess the degree of joint swelling at 6 weeks after the operation. The results indicated that MB treatment obviously helped relieve joint swelling during OA progression (Fig. 1c). Moreover, gait analysis clearly revealed claudication during walking due to pain. The prints of the front and hind paws of a healthy rat or presurgical rat were relatively overlapping, while those of the rats in the OA group were obviously separated; this change was significantly more severe (indicated by a greater distance between the prints of the front and hind paws) in the OA group than in the OA + MB group. The average stride length (blue dotted line) and step length (red dotted line) of the OA + MB group were more similar to those of the sham group than to those of the OA group (Fig. 1d, e). The results of gait analysis further validated that MB treatment relieved pain. Histological analysis, which involved safranin O and toluidine blue staining and grading of cartilage and osteophytes according to the OARSI scoring system, showed that the OA + MB group exhibited less proteoglycan loss and smaller osteophytes than the OA group (Fig. 1f–h). H&E staining and synovitis scores indicated the presence of synovial hypercellularity and thickening in the OA group, whereas MB treatment alleviated synovitis (Fig. 1f, j). Micro-CT analysis further confirmed that the MB treatment group presented less osteophyte (indicated by red arrows) formation than the OA group (Fig. 1i).

### MB reduces TBHP-induced metalloproteinase expression and oxidative stress in hCHs and hFLSs

**Fig. 2:**
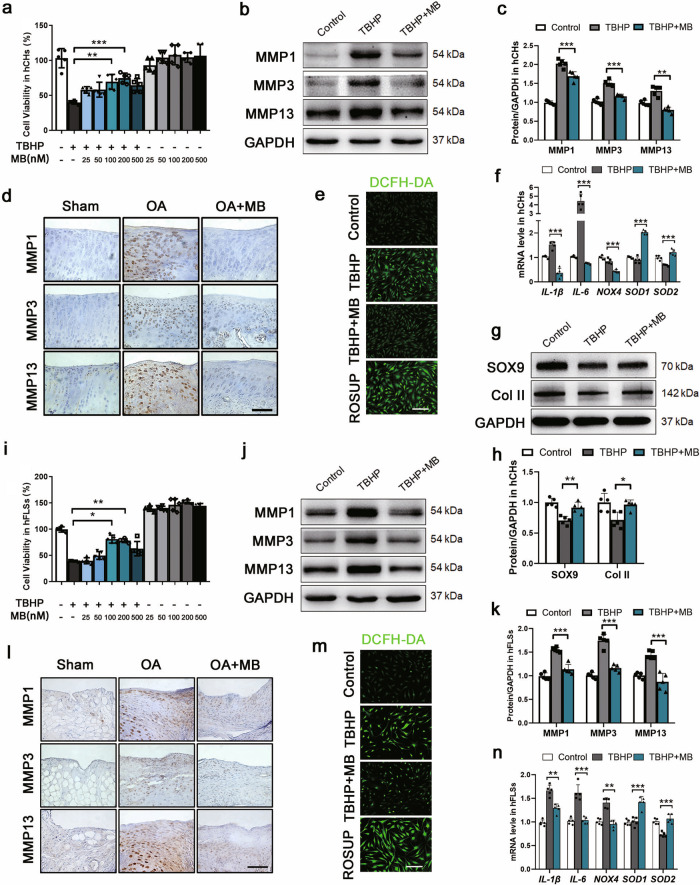
MB reduces TBHP-induced metalloproteinase expression and oxidative stress in hCHs and hFLSs. **a**, **i** The viability of human OA chondrocytes (hCHs) and human OA fibroblast-like synoviocytes (hFLSs) after TBHP treatment and treatment with or without MB. **b**, **j** Representative Western blot results for MMP1, MMP3, MMP13 in hCHs and hFLSs after TBHP treatment and treatment with or without MB. Quantification of the Western blot data from **b** (**c**) and **j** (**k**). Immunohistochemical staining for MMP1, MMP3, and MMP13 in the affected joint cartilage (**d**) and synovium (**l**) of rats (scale bar: 100 μm). Fluorescence images of the DCFH-DA probe for hydrogen peroxide in hCHs (**e**) and hFLSs (**m**) in each group (scale bar: 100 μm). **f**, **n** Quantitative real-time PCR analysis of *IL-1β, IL-6, NOX4, SOD1*, and *SOD2* mRNA levels in hCHs and hFLSs. **g** Representative Western blot results for SOX9 and Col II in hCHs after TBHP treatment and treatment with or without MB. **h** Quantification of the Western blot data from **h**. All data represent the mean ± S.D. (*n* = 5). *P < 0.05, **P < 0.01, and ***P < 0.001.

The cytotoxic effects of MB on the viability of human OA chondrocytes (hCHs) and fibroblast-like synoviocytes (hFLSs) were assessed by CCK-8 assay. MB treatment had no significant cytotoxic effect on hCHs and even slightly induced the proliferation of hFLSs when administered at concentrations between 25 nM and 500 nM for 6 h (Fig. 2a, i). Tert-butyl hydroperoxide (TBHP) was utilized to induce oxidative stress. The viability of hCHs and hFLSs was decreased by more than 50% upon stimulation with TBHP (100 μM) for 6 h. However, this cytotoxicity was significantly attenuated by MB (200 nM and 100 nM) in hCHs and hFLSs. Western blot analysis of hCHs and hFLSs stimulated with TBHP and immunohistochemical staining of the cartilage and synovia of rats with OA showed that the expression of metalloproteinases (MMPs), including MMP 1, 3, 13, was significantly increased by MB treatment (Fig. 2b–d, j–i). 2′,7′-Dichlorodihydrofluorescein diacetate (DCFH-DA) was used to evaluate the level of intracellular ROS. Fluorescence analysis showed a markedly lower density of ROS in hCHs and hFLSs in the TBHP + MB group than in the TBHP group and the positive control (ROSUP) group (Fig. 2e, m). Moreover, the mRNA levels of inflammatory factors, including interleukin 1β (*IL-1β*) and *IL-6*, and oxidative stress products, such as NADPH oxidase 4 (*NOX4*), were apparently reduced following MB treatment compared with TBHP treatment alone, while superoxide dismutase1 (*SOD1*) and superoxide dismutase2 (*SOD2*) levels were increased (Fig. 2f, n). Furthermore, Western blot analysis of SOX9 and Collagen II (Col II) expression revealed that the loss of chondrocytic activity in hCHs induced by TBHP was alleviated by MB treatment (Fig. 2g, h). These results showed that MB had anti-inflammatory and antioxidative stress effects in hCHs and hFLSs.

### MB increases the expression and translocation of Nrf2 in hCHs and hFLSs

**Fig. 3:**
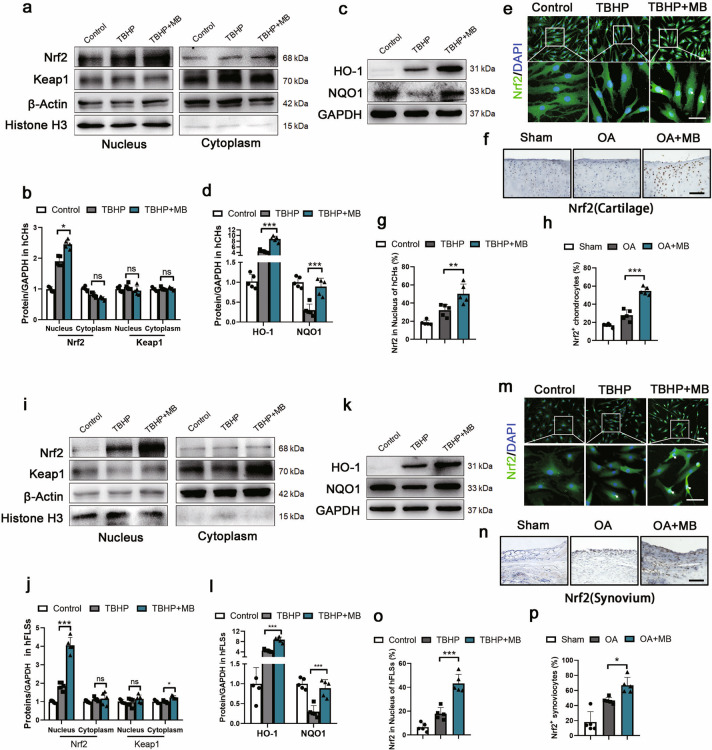
MB increases the expression and nuclear translocation of Nrf2 in hCHs and hFLSs. Representative Western blot results for nuclear and cytoplasmic expression of Nrf2 and Keap1 in hCHs and hFLSs after TBHP treatment and treatment with or without MB. **b**, **j** Quantification of Western blot data from **a** (**b**) and **i** (**j**). Representative Western blot results for HO-1 and NQO1 in hCHs and hFLSs after TBHP treatment and treatment with or without MB. Quantification of the Western blot data from **c** (**d**) and **k** (**l**). Immunofluorescence staining for Nrf2 in hCHs and hFLSs (green: Nrf2;blue: DAPI; scale bar: 100 μm). **f**, **n** Immunohistochemical staining for Nrf2 in the affected joint cartilage and synovium of rats (scale bar: 100 μm). Quantification of the immunofluorescence staining data from **e** (**g**) and **m** (**o**). **h**, **p** Quantification of the immunohistochemical staining data from **f** (**h**) and **n** (**p**). All data represent the mean± S.D. (*n* = 5). *P < 0.05, **P < 0.01, and ***P < 0.001.

To investigate the underlying mechanism of the antioxidative stress effect of MB, we first evaluated the expression of Nrf2 in hCHs and hFLSs treated with TBHP and with or without MB. Nrf2 synthesizes and accumulates in the cytoplasm and needs to translocate to the nucleus to induce the expression of its target phase II genes, such as oxygenase-1 (HO-1), quinone oxidoreductase 1 (NQO1), and PRDX1 [22, 32]. Kelch-like ECH-associated protein 1 (Keap1) interacts with Nrf2 to form a complex that serves as the major negative regulator of Nrf2 activity [33]. Therefore, we separated nuclear and cytoplasmic proteins and measured the expression of Nrf2 and Keap1 via Western blot analysis. The results showed that Nrf2 expression was markedly upregulated in the nucleus following stimulation with TBHP and that this increase in expression was further enhanced by MB treatment however no significant difference in expression was observed in the cytoplasm (Fig. 3a, b, i, j). Moreover, there was no significant difference in the expression of Keap1 in either the nucleus or cytoplasm in hCHs (Fig. 3a, b). Keap1 expression in hFLSs showed an increasing trend in the MB + TBHP group in the cytoplasm but not in nucleus (Fig. 3i, j). Moreover, the expression of proteins downstream of Nrf2, such as HO-1 and NQO1, was increased in hCHs and hFLSs treated with MB (Fig. 3c, d, k, l). Immunofluorescence staining of hCHs and hFLSs clearly showed that the density of Nrf2 in the nucleus was higher in the TBHP + MB group than in the TBHP group and control group (Fig. 3e, g, m, o). We employed immunohistochemical analysis to compare Nrf2 levels in rats with OA treated with or without MB and sham rats. Similar to what was found in vitro, significant enhancement of Nrf2 expression was observed in the OA + MB group in both the cartilage and synovium (Fig. 3f, h, n, p). Together, these results indicated that the MB exerted its antioxidative stress effect by increasing the expression of Nrf2 and promoting its translocation to the nucleus.

### MB increases the expression PRDX1 in hCHs and hFLSs

**Fig. 4:**
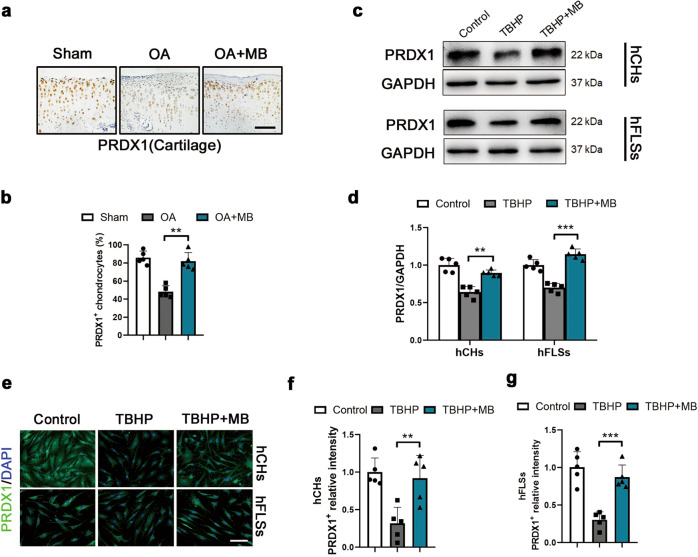
MB increases the expression PRDX1 in hCHs and hFLSs. **a** Immunohistochemical staining for PRDX1 in the affected joint cartilage of rats (scale bar: 100 μm). **b** Quantification of the immunohistochemical staining data from **a**. **c** Representative Western blot results for PRDX1 in hCHs and hFLSs after TBHP treatment and treatment with or without MB treatment. **d** Quantification of the Western blot data from **c**. **e** Representative immunofluorescence staining for PRDX1 in hCHs and hFLSs (green: PRDX1; blue: DAPI; scale bar: 100 μm). **f, g** Quantification of the immunofluorescence staining data from **e**. All data represent the mean ± S.D. (*n* = 5). *P < 0.05, **P < 0.01, and ***P < 0.001.

To explore the precise mechanism underlying the antioxidative stress effect of MB, we studied PRDX1, an Nrf2-dependent phase II gene, which is a vital antioxidant enzyme that has not been studied in the context of OA. Immunohistochemical analysis was performed to measure the expression level of PRDX1 in the articular cartilage of rats with OA. Immunohistochemical staining revealed a significant decrease in PRDX1 expression in chondrocytes in the OA group compared with the sham group, which was apparently ameliorated by MB treatment (Fig. 4a, b). In vitro, the protein expression of PRDX1 was downregulated after oxidative stress injury induced by TBHP treatment but was maintained at a high level in both hCHs and hFLSs in the TBHP + MB group (Fig. 4c, d). As shown by immunofluorescence staining for PRDX1, PRDX1 expression was increased in hCHs and hFLSs in the TBHP + MB group compared with the TBHP group (Fig. 4e–g). Herein, we concluded that excessive oxidative stress inhibited PRDX1 expression but that MB prevented this negative effect and restored the level of PRDX1.

### MB regulates the interaction between Nrf2 and PRDX1 under oxidative stress

**Fig. 5:**
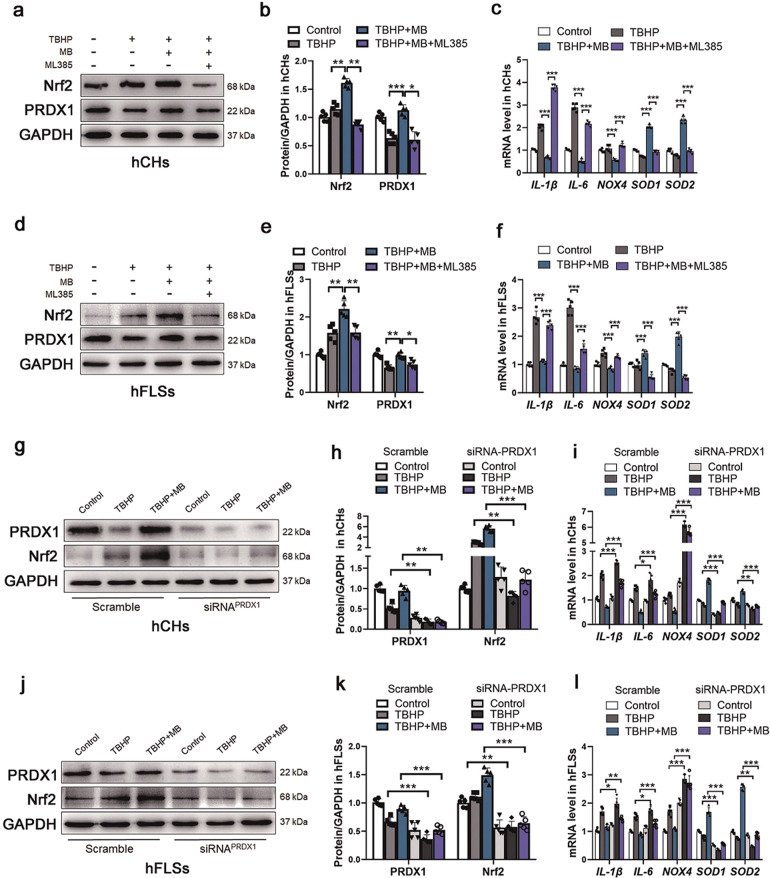
The relationship between Nrf2 and PRDX1 under oxidative stress. **a**, **d** Representative Western blot results for Nrf2 and PRDX1 in hCHs and hFLSs after TBHP treatment, treatment with or without MB treatment, and treatment with or without ML385 (an Nrf2 inhibitor). Quantification of the Western blot data from **a** (**b**) and **d** (**e**). **c**, **f** Quantitative real-time PCR analysis of *IL-1β, IL-6, NOX4, SOD1,* and *SOD2* mRNA levels in hCHs and hFLSs after TBHP treatment, treatment with or without MB treatment, and treatment with or without ML385 (an Nrf2 inhibitor). **g**, **j** Representative Western blot results for Nrf2 and PRDX1 in hCHs and hFLSs transfected with PRDX1-siRNA after TBHP treatment and treatment with or without MB. **h**, **k** Quantification of the Western blot data from **g** (**h**) and **j** (**k**). **i**, **l** Quantitative real-time PCR analysis of *IL-1β, IL-6, NOX4, SOD1*, and *SOD2* mRNA levels in hCHs and hFLSs after TBHP treatment and treatment with or without MB. All data represent the mean ± S.D. (*n* = 5). *P < 0.05, **P < 0.01, and ***P < 0.001.

**Fig. 6:**
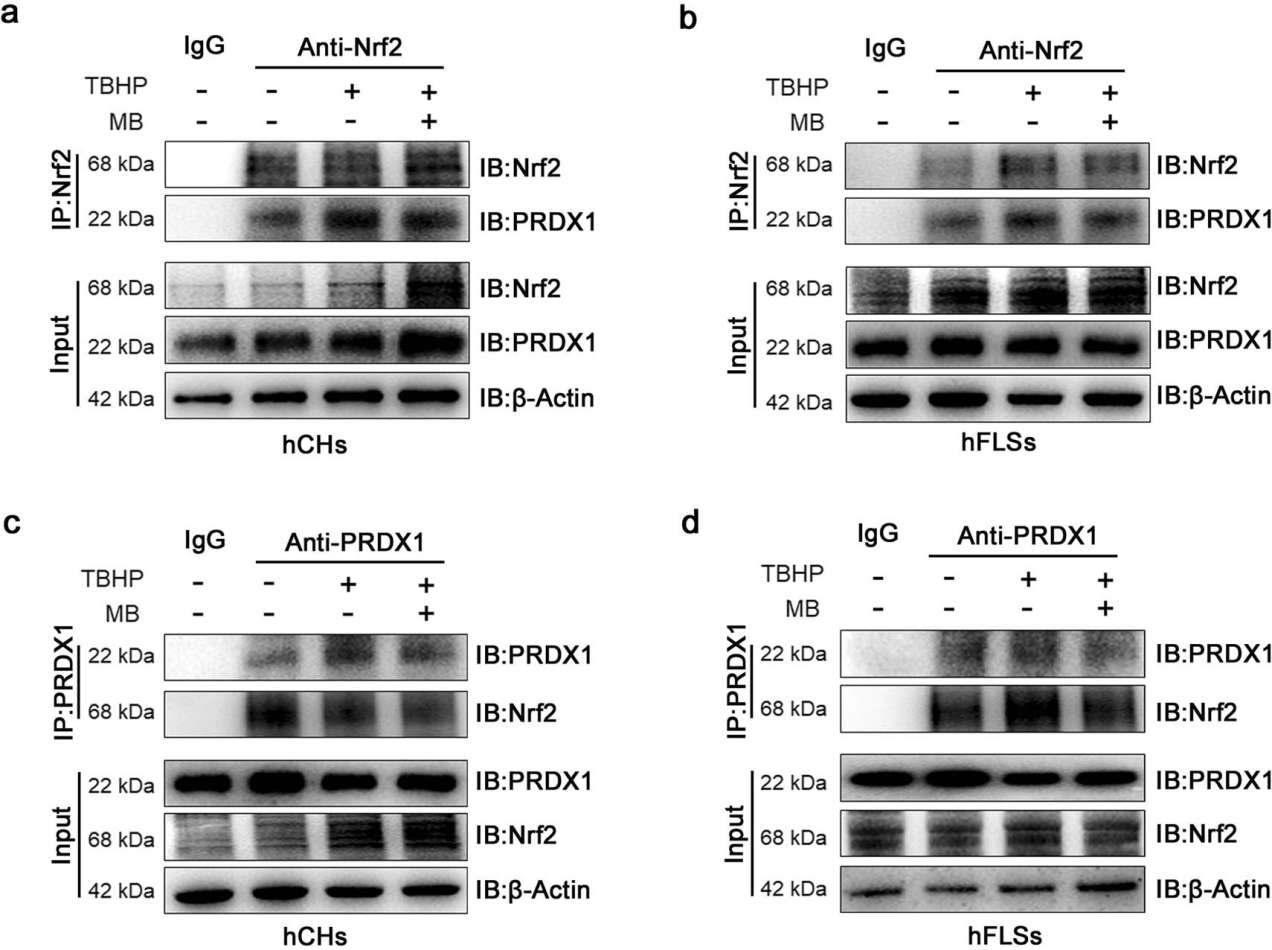
Binding between Nrf2 and PRDX1 under oxidative stress. **a**, **b** Representative coimmunoprecipitation analysis of the binding of PRDX1 to Nrf2 in hCHs and hFLSs after TBHP treatment and treatment with or without MB. **c**, **d** Representative coimmunoprecipitation analysis of the binding of Nrf2 to PRDX1 in hCHs and hFLSs after TBHP treatment and treatment with or without MB.

To study the exact relationship between Nrf2 and PRDX1 in hCHs and hFLSs under oxidative stress in the presence of MB, we expression levels of Nrf2 and PRDX1 after treatment with ML385 (an Nrf2 inhibitor) and transfection of an siRNA targeting PRDX1, respectively. ML385 was identified as a specific probe molecule that binds to Neh1, the cap “*n*” collar basic leucine zipper domain of Nrf2, and interferes with the binding of the V-Maf musculoaponeurotic fibrosarcoma oncogene homolog G (MAFG)-NRF2 protein complex to regulatory DNA binding sequences [34]. As shown in Fig. 5a, b, d, e, we first analyzed Nrf2 and PRDX1 expression in hCHs and hFLSs by Western blotting following treatment with ML385 in vitro. The total protein expression of Nrf2 was increased in the group treated with TBHP and MB simultaneously compared to the group treated with TBHP alone and markedly inhibited by the addition with ML385, even when oxidative stress was reduced by treatment with MB. Moreover, consistent with the suppression of Nrf2 expression was suppressed, the effect of MB in increasing PRDX1 expression was inhibited by ML385, which demonstrated that Nrf2 has a positive regulatory relationship with PRDX1. Moreover, the inhibitory effect of MB on *IL-1β*, *IL-6*, and *NOX4* expression and its promoting effect on *SOD1* and *SOD2* expression were impaired in hCHs and hFLSs (Fig. 5c, f). Then, we transfected an siRNA against PRDX1 into hCHs and hFLSs under oxidative stress in the presence or absence of MB. The level of PRDX1 was obviously decreased after transfection with siRNA, and no significant difference was observed between the TBHP group and TBHP + MB group. Furthermore, the expression level of Nrf2 was similar to that of PRDX1, and Nrf2 expression was inhibited even following the addition of MB (Fig. 5g, h, j, k). Moreover, the effects of MB on *IL-1β, IL-6, NOX4, SOD1*, and *SOD2* expression were inhibited by PRDX1 knockdown (Fig. 5i, l). To assess the binding between Nrf2 and PRDX1, coimmunoprecipitation was performed, and the results indicated that Nrf2 was coupled with PRDX1 and that there was no significant change in the binding of these proteins between the TBHP group and the TBHP + MB group (Fig. 6a–d). Considering these data, we concluded that there was an interaction between Nrf2 and PRDX1 and that MB was unable to exert its full antioxidative effect when one of these proteins was inhibited.

### MB inhibits CGRP in neurons by regulating Nrf2 and PRDX1 under oxidative stress

**Fig. 7:**
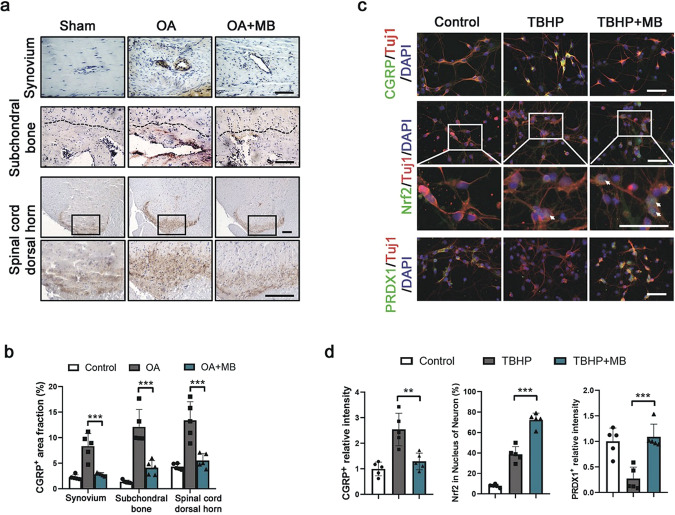
MB inhibits CGRP and regulates Nrf2 and PRDX1 under oxidative stress in neurons. **a** Immunohistochemical staining for CGRP in the affected joint synovium, subchondral bone and spinal cord dorsal horn (L3-L5) in rats (scale bar: 100 μm). The black dotted line represents the boundary between cartilage and subchondral bone. The subchondral bone is below this boundary. **b** Quantification of the immunohistochemical staining data from **a**. **c** Double immunofluorescence staining for TUJ1 and CGRP in primary neurons (red: TUJ1; green: CGRP; blue: DAPI; scale bar: 100 μm), TUJ1 and Nrf2 in primary neurons (red: TUJ1; green: Nrf2; blue: DAPI; scale bar: 100 μm), and TUJ1 and PRDX1 in primary neurons (red: TUJ1; green: PRDX1; blue: DAPI; scale bar: 100 μm). **d** Quantification of the immunofluorescence staining data from **c**. All data represent the mean ± S.D. (*n* = 5). *P < 0.05, **P < 0.01, and ***P < 0.001.

To explore the mechanism of the pain-relieving effect of MB, we studied calcitonin gene-related peptide (CGRP), which is released from the endings of sensory and efferent nerves, is considered a major component of neurogenic inflammation and is related to several clinical pain syndromes [35, 36]. The results of the immunohistochemical analysis showed that CGRP accumulated around the vessels in the synovium and subchondral bone in animals with OA and that CGRP levels were markedly reduced after MB treatment. Interestingly, CGRP was also expressed in the dorsal horn of the lumbar spinal cord (L3-L5) of rats, and CGRP expression increased with OA development but decreased upon administration of MB (Fig. 7a, b). Moreover, we examined the effect of MB treatment on oxidative stress in primary neurons. Double immunofluorescence staining for CGRP and TUJ-1 (β-III-tubulin, a protein marker of neurons) was performed after neurons were treated with TBHP and with or without MB for 4 h. The results showed that CGRP was remarkably enriched in neurons in the TBHP group but not in the control group or TBHP + MB group (Fig. 5c, d). These data indicated that MB inhibited the synthesis and release of CGRP. Furthermore, as shown in Fig. 5c, d, Nrf2 was more enriched in the nuclei of neurons treated with both MB and TBHP than in the control and TBHP groups. Staining for PRDX1 showed that oxidative stress induced by TBHP significantly reduced the level of PRDX1 in neurons but that PRDX1 expression was obviously increased by MB (Fig. 5c, d). Taken together, these findings indicated that MB also enhanced the regulation of Nrf2 and PRDX1 to inhibit CGRP, which contributes to pain and neuroinflammation, in neurons.

## Discussion

The roles of oxidative stress have been increasingly researched, and oxidative stress is recognized as being closely related to OA pathology and development. At the cellular level, excessive oxidative stress leads to severe mitochondrial DNA (mtDNA) and nuclear DNA damage, influencing cell signaling pathways and protein transcription [37]. Excessive oxidative stress not only causes aberrations in bone and cartilage metabolism, leading to structural degradation but also stimulates cells residing in the synovium, such as synoviocytes and peripheral nerve cells, which contribute to the major symptoms of OA, pain, and arthrocele [38]. When OA occurs, excessive oxidative stress can cause changes in the homeostasis of the original microenvironment, such as abnormal ROS release, accumulation of MMPs, and impairment of cellular functions. Therefore, we identified scavenging of oxygen free radicals and amelioration of oxidative stress as important strategies for preventing OA progression and treating the disease.

It has been demonstrated that the pain associated with OA is nociceptive pain, allodynia, and hyperalgesia coupled with local inflammation and neuropathic processes in the joint [39]. MB has a strong affinity for nerves, and previous studies have demonstrated that MB can be utilized to treat clinical pain; but the effect of MB in OA has not yet been studied. In this study, the intra-articular injection of MB into OA model rats significantly ameliorated arthrocele of the affected knee joint and behavioral alterations, including changes in weight distribution and the PWMT of the affected limb. We performed gait analysis to intuitively assess the pain behavior of OA model rats. As shown in Fig. 1d, e, the separation of the prints of the hind and forepaws on the affected side and the changes in step length and stride length clearly reflected claudication of the rats during walking. According to the results, the severity of claudication was obviously relieved by MB treatment. In addition, we demonstrated that MB protected cartilage and prevented osteophyte formation during OA and exerted anti-inflammatory and antioxidative stress effects in hCHs and hFLSs in vitro. Cartilage loss, synovitis, and pain are hallmarks of OA progression in the clinic and the main therapeutic targets of recent research on OA treatment [1, 40]. Our results showed that MB could not only protect against structural damage specific to OA but also significantly relieve pain-related symptoms. Furthermore, research in hCHs and hFLSs revealed that MB had excellent antioxidant effects against cartilage loss and synovitis. We believe that these effects of MB fully and effectively protect damaged joints in OA.

The oxidant-antioxidant balance is one of the key factors in maintaining the health of the cartilage and synovium, and disruption of this balance is thought to contribute to inflammation in the initiation and progression of OA. A number of enzyme systems and molecular factors are involved in antioxidant processes that prevent OA progression. In this study, it was demonstrated that the expression of Nrf2 and its translocation to the nucleus were both increased via MB treatment in chondrocytes and synoviocytes. Keap1 is considered a major negative regulator of Nrf2 activity and serves as the intracellular sensor of electrophiles and oxidants, which induce stabilization of Nrf2 in the cytoplasm [33, 41]. We found that MB did not have a significant effect on the expression of Keap1, which indicated that MB did not affect Nrf2 through Keap1 in vitro. Moreover, Nrf2 not only inhibits inflammation through redox regulation but also downregulates the expression of chemokines, proinflammatory cytokines, adhesion enzymes, and molecules [42]. However, MB has been reported to upregulate Nrf2 expression when used to treat Tau protein-related neurotoxicity [19]. This study is the first to report the regulatory effect of MB on Nrf2 in OA and to describe the strong ability of MB to resist cartilage degeneration, inflammation, and oxidative stress in OA development and pain. The Nrf2-dependent expression of phase II genes, including HO-1, NQO1, and PRDX1, plays important roles in maintaining redox homeostasis and regulating the inflammatory response [43]. We observed that the expression levels of HO-1, NQO1, and PRDX1 were upregulated by MB treatment in the presence of TBHP. The effect of HO-1 and NQO1 against ROS in OA has been widely reported [44]; however, the effect of PRDX1 on ROS in OA has not yet been reported. Several studies have demonstrated that PRDX1 and signaling pathways involved in PRDX1-mediated regulation of ROS play a crucial role in the progression and metastasis of human tumors [45, 46]. Although PRDX1 has been shown to exert strong antioxidant effects in cancer treatment, its effects in OA have not yet been studied. We first assessed the expression of PRDX1 in OA development, and the results showed that PRDX1 was expressed both in vivo and in vitro. PRDX1 expression decreased with disease progression and increased upon treatment with MB. PRDX1 cooperates with the thioredoxin (Trx) system to suppress H_2_O_2_-induced cell death involving several kinases and enzymes that play vital roles in the regulation of cell death and/or apoptosis [23, 47, 48]. In addition, we administered an inhibitor of Nrf2 to and transfected a siRNA against PRDX1 into hCHs and hFLSs and observed that Nrf2 and PRDX1 promoted the expression of the other and worked closely together upon the administration of MB, which is consistent with previous research showing that Nrf2 and PRDX1 work together to regulate prostaglandin D2 and E2 production in macrophages in the context of acute inflammation [24]. Moreover, it has been reported that overexpression of PRDX1 increases the levels of Nrf2 and its downstream antioxidant protein [49]. To confirm that Nrf2 and PRDX1 interact, we performed coimmunoprecipitation and found that Nrf2 and PRDX1 directly bound to each other. This result suggested that MB targeted two antioxidant signaling pathways, i.e., as the Nrf2 and PRDX1 pathways, to resist oxidative stress and thus prevent OA progression and pain and that MB may have a positive feedback effect on these pathways.

As mentioned above, in addition to having antioxidant capacity, MB has another interesting characteristic: it exhibits a great affinity for neurons without inducing pathologic changes and has a remarkable ability to permeate bio-membranes due to its high lipophilicity [11, 50, 51]. In research on ischemia in rats, MB was demonstrated to play a dual role, having a potent analgesic effect at lower doses [52]. The precise mechanisms underlying these effects of MB are still not completely understood. It has been reported that inflammatory mediators, including bradykinin, substance P, histamine, and CGRP, induce hyperalgesia by many direct and/or indirect actions and trigger the release of additional inflammatory mediators [53, 54]. In this study, we demonstrated that CGRP accumulated around the vasculature in the rat synovium, subchondral bone, and spinal cord dorsal horn (L3-L5) when OA was induced, and that CGRP accumulation was significantly reduced by MB treatment. Peripheral sensitization is important in the development and maintenance of central sensitization. Intense, repeated, or prolonged signals from peripheral nociceptors regulate spinal cord pain-transmitting neurons and lead to decreased activation thresholds and increased synaptic excitability and firing thresholds [55, 56]. This explains why when OA occurs in the knee joint, pain-related molecular changes can be detected in both the knee synovium and the central spinal cord. Furthermore, immunofluorescence staining of primary neurons showed that the expression of CGRP was enhanced under TBHP stimulation and reduced by the addition of MB. We also observed that MB treatment enhanced the expression of Nrf2 and PRDX1 in primary neurons via immunofluorescence staining. Thus, we speculated that the analgesic effect of MB and the inhibitory effect on neuroinflammatory substances such as CGRP may result from the activation of the Nrf2/PRDX1 signaling pathways in primary neurons. However, the precise mechanism involved in the effect of MB on pain-related neurotransmitters and neuroinflammation remains elusive and needs to be further researched.

**Fig. 8:**
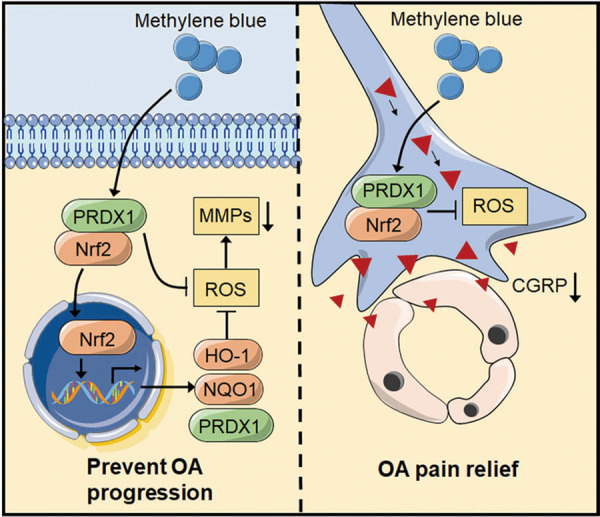
A schematic showing that methylene blue ameliorates oxidative stress by regulating Nrf2/PRDX1 to prevent disease progression and relieve pain in osteoarthritis.

To summarize, the results suggested that the MB prevented OA progression and pain by exerting an efficient antioxidant effect associated with the promotion of the Nrf2/PRDX1 signaling pathway (Fig. 8). In addition, we demonstrated targeting both Nrf2 and PRDX1 promoted the expression of both of these molecules to further amplify the therapeutic effect of MB against OA. In this study, MB protected cartilage and exerted anti-inflammatory effects in the synovium and neurons. However, there are unanswered questions that need to be resolved by further studies. For example, the optimal molecular target and route of administration for interfering with Nrf2/PRDX1 are unclear, and the detailed relationship between Nrf2 and PRDX1 is not completely understood. Moreover, intra-articular delivery and release of MB should be combined with cartilage regeneration strategies to improve drug utilization and disease treatment. To conclude, our results show that MB is an effective agent for OA therapy and elucidate the precise mechanism of its antioxidative effect in OA. This work provides a theoretical basis for the therapeutic effect of MB in preventing disease progression and pain in patients with OA.
